# Supplementary material for: Nuclear translocation of mitochondrial dehydrogenases as an adaptive cardioprotective mechanism
Source: Nat Commun. 2023 Jul 19;14:4360. doi: 10.1038/s41467-023-40084-5 (PMC10356764; doi:10.1038/s41467-023-40084-5)
Supplement: Supplementary file 1 — Supplementary Information [file 41467_2023_40084_MOESM1_ESM.pdf]

Supplementary Fig.1

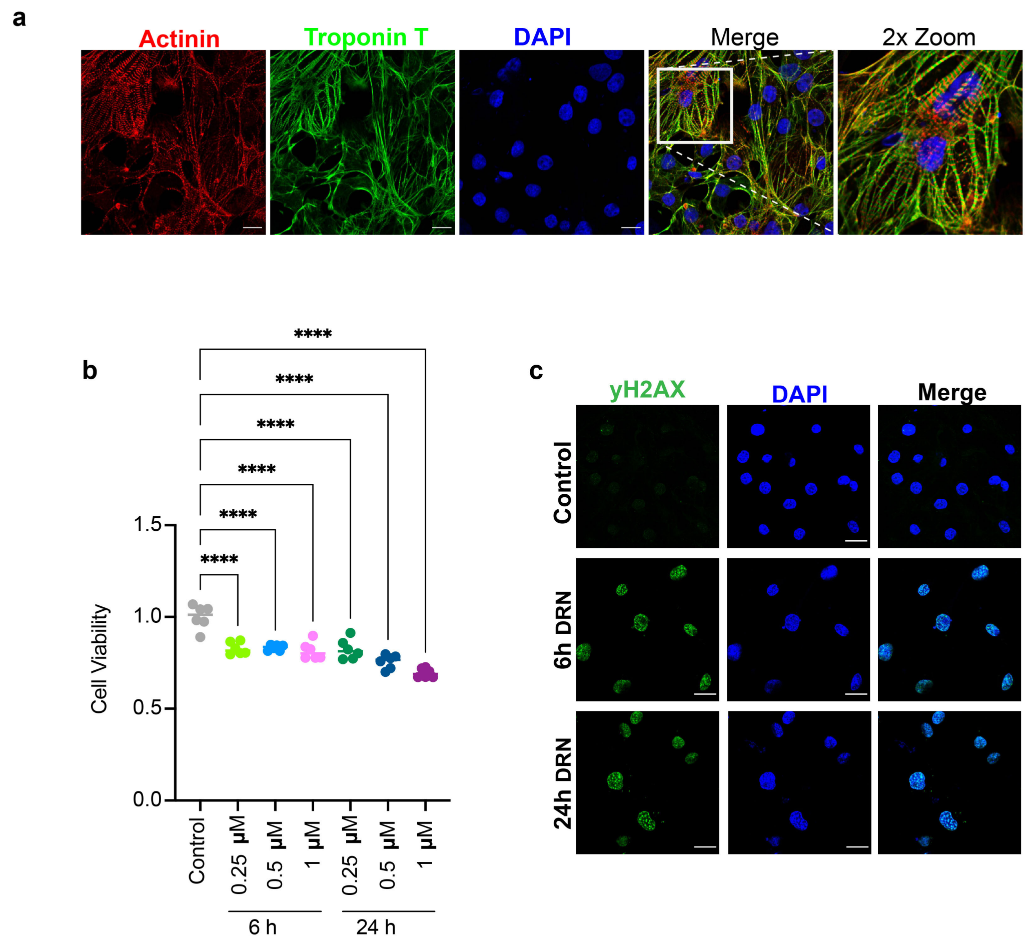

Supplementary Fig.1: (a) hiPSC-derived cardiomyocytes stained with cardiomyocytes markers Actinin and Troponin T which were detected by alexa-594 and alexa-488 respectively, nuclei were stained with DAPI. scale bar 20 µm. (b) Cardiomyocytes were treated with increasing concentration of doxorubicin (DRN) (0.25 µM to 1 µM) for 6h or for 24 h and cell death was measured by Cell titer glow. p-value****<0.0001. (c) yH2AX foci formation measured as an indicator of DNA double strand breaks in iPSC-derived cardiomyocytes treated with 1 µM DRN. The foci were detected with anti- yH2AX antibody with secondary antibody conjugated with alexa-488.
scale bar 20 µm.

Supplementary Fig.2

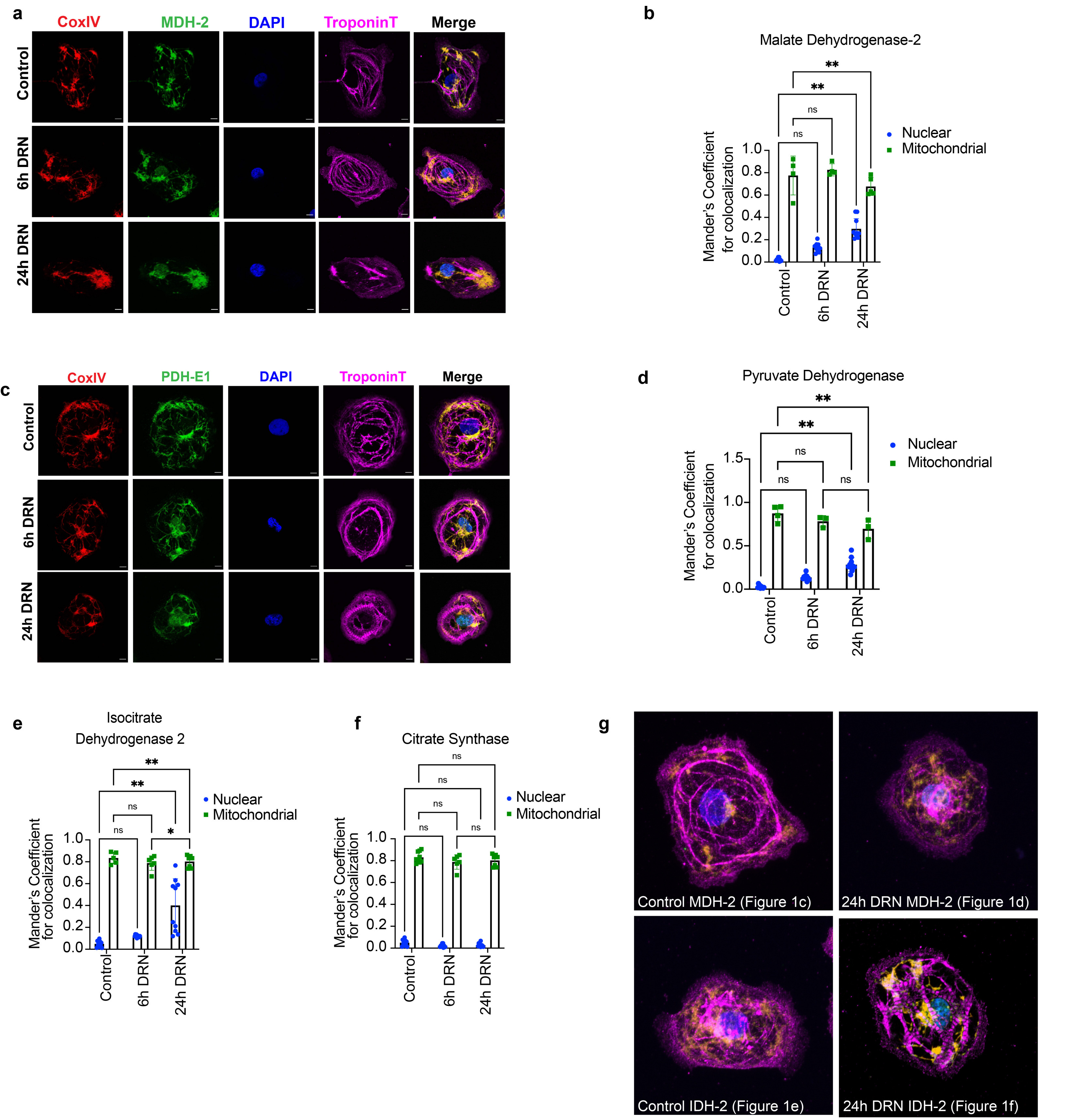

Supplementary Fig.2: **(a, c)** Confocal images showing subcellular localization of MDH-2 **(a)** and PDH-E1 **(c)** in iPSC-CM upon doxorubicin treatment. DAPI and CoxIV were used to counterstain nucleus and mitochondria respectively, and TroponinT was used as cardiomycocyte marker. **(b, d)** Quantification of mitochondrial and nuclear localization of MDH-2 **(b)** and PDH-E1 **(d)** p-value**<0.001.
(e,f) Quantification of images represented in figure 1a **(e)** and 1b **(f)**. scale bar 20 μm. All the image quantification was done using JACaP plugin on ImageJ/Fiji to quatify the co-localization of TCA cycle dehydrogenases (green) with nuclei (DAPI, blue) and represented as Mander's co-efficient of co-localization. **(g)** Troponin T staining of cells shown in figure 1c-f. Image shown in panel g here (right hand lower most corner) and in fig.1f are also shown in fig.1a (lower most images)

Supplementary Fig.3

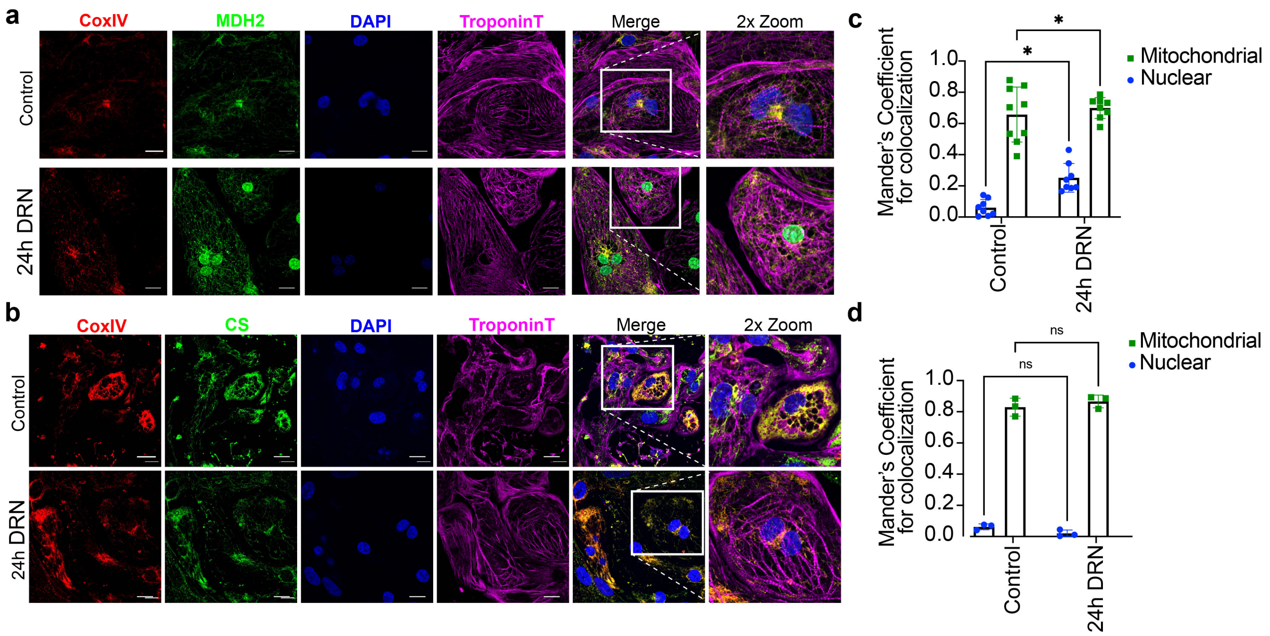

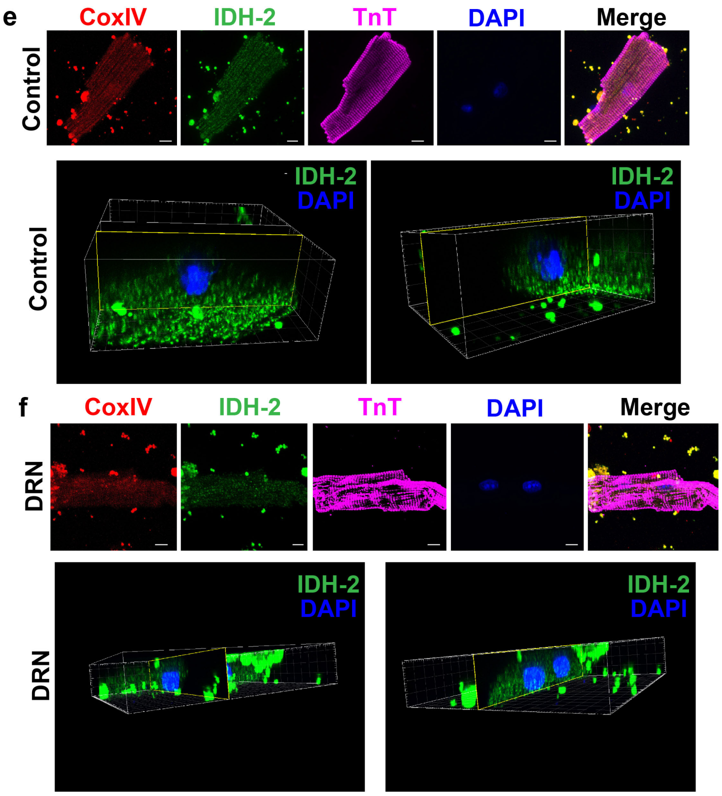

Supplementary Fig.3: (a, b) Immunofluoroscent images of 60 days old comaparatively mature cardiomyocytes treated with doxorubicin (DRN) for 24h. Cells were stained with MDH-2 **(a)** and CS **(b)**, and detected with secondary antibody conjugated with alexa-594. CoxIV and TnT were stained as markers of mitochondria and cardiomyocytes. scale bar 20µm. **(c, d)** Quantification of MDH-2 **(c)** and CS **(d)** in images A and B respectively. p value*<0.05.
(e-f) Cardiomyocytes isolated from control mouse **(e)** and mouse treated with DRN **(f)**. Inages show the images presented in figure 1h-i with TroponinT as cardiomyocyte-specific marker. scale bar: 10 µm

Supplementary Fig.4

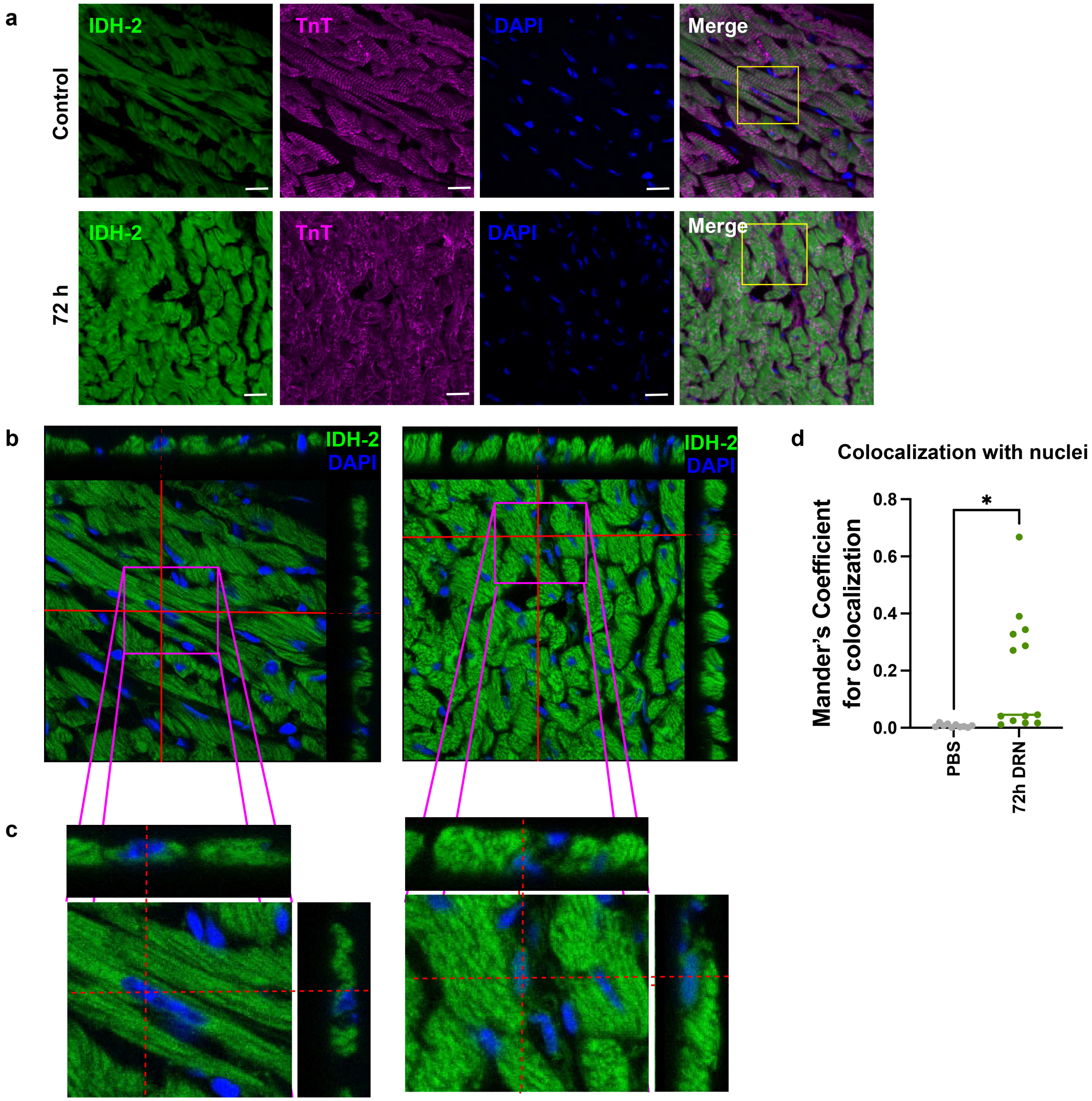

Supplementary Fig.4: **(a)** Maximum Projection Intensity of z-stack of cardiac tissue sections stained with Troponin T (TnT, magenta), IDH-2 (Green), and DAPI (nuclei, DAPI) from heart tissue isolated from PBS or DRN treated mice. scale bar 5 µm **(b)** Orthogonal section of zstack of images shown in panel A. The red cross lines indicate the nucleus of interest highlighted to show nuclear presence of IDH-2 in DRN treated animal.
(c) Enlarged images from panel B. Yellow box in panel A shows that the nuclei shown in panel B (pink box) and enlarged in panel C correspond to TnT positive cardiomyocytes within the tissue section. **(d)** Quantification of IDH-2 nuclear localization in tissue sections from animals treated with PBS or DRN for 72h. p value=0.0107

Supplementary Fig.5

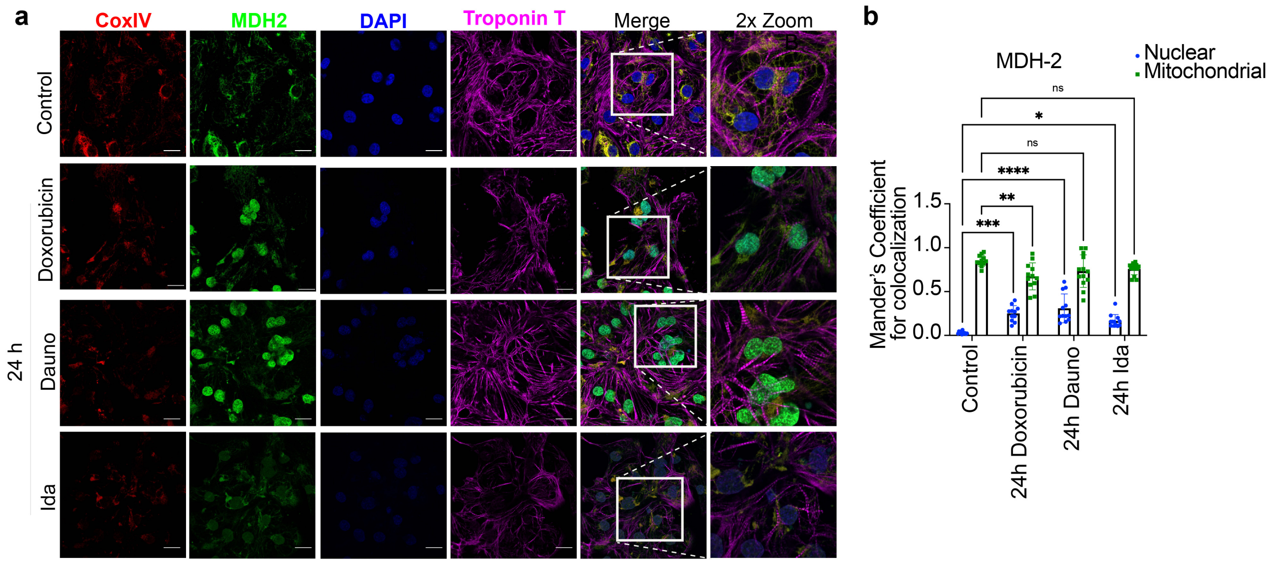

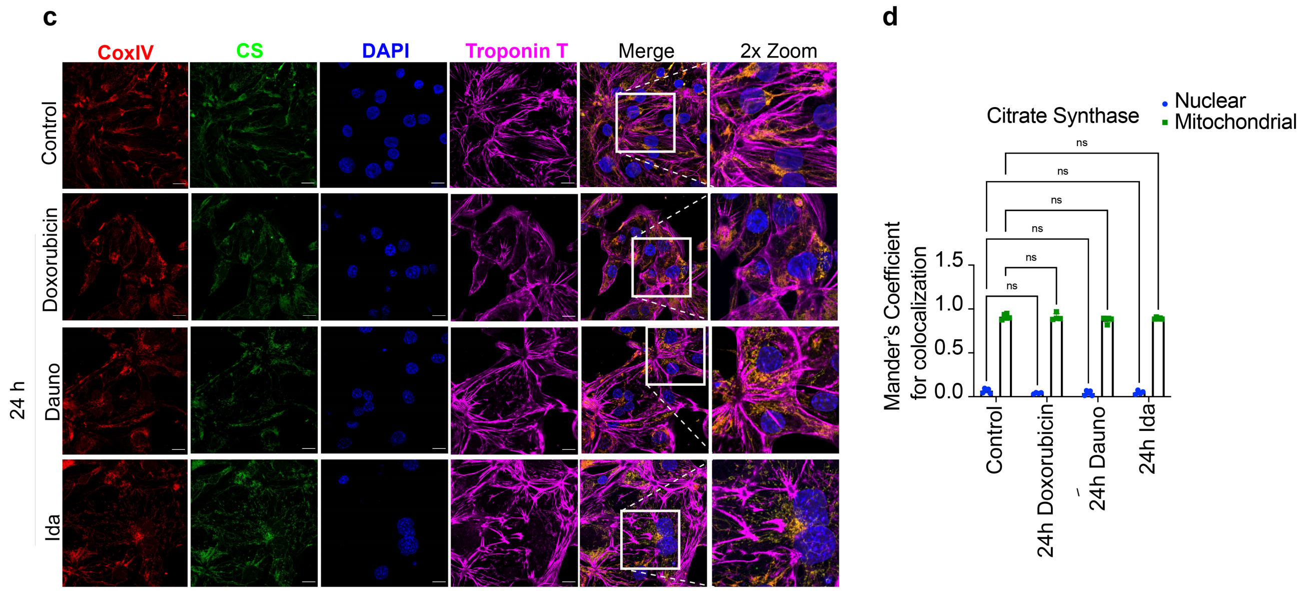

Supplementary Fig.5: Immunofluoroscent images of cells treated with different anthracyclines: doxorubicin, daunorubicin, and idarubicin for 24h. Cells were stained with MDH-2 **(a)** and CS **(c)**, and detected with secondary antibody conjugated with alexa-594. CoxIV and TnT were stained as markers of mitochondria and cardiomyocytes. scale bar 20μm. Quantification of MDH-2 **(b)** and CS **(d)** in images a and c respectively. p-value*=0.0376, p-value**=0.0024, p-value***=0.0002, p-value****<0.0001.

Supplementary Fig.6

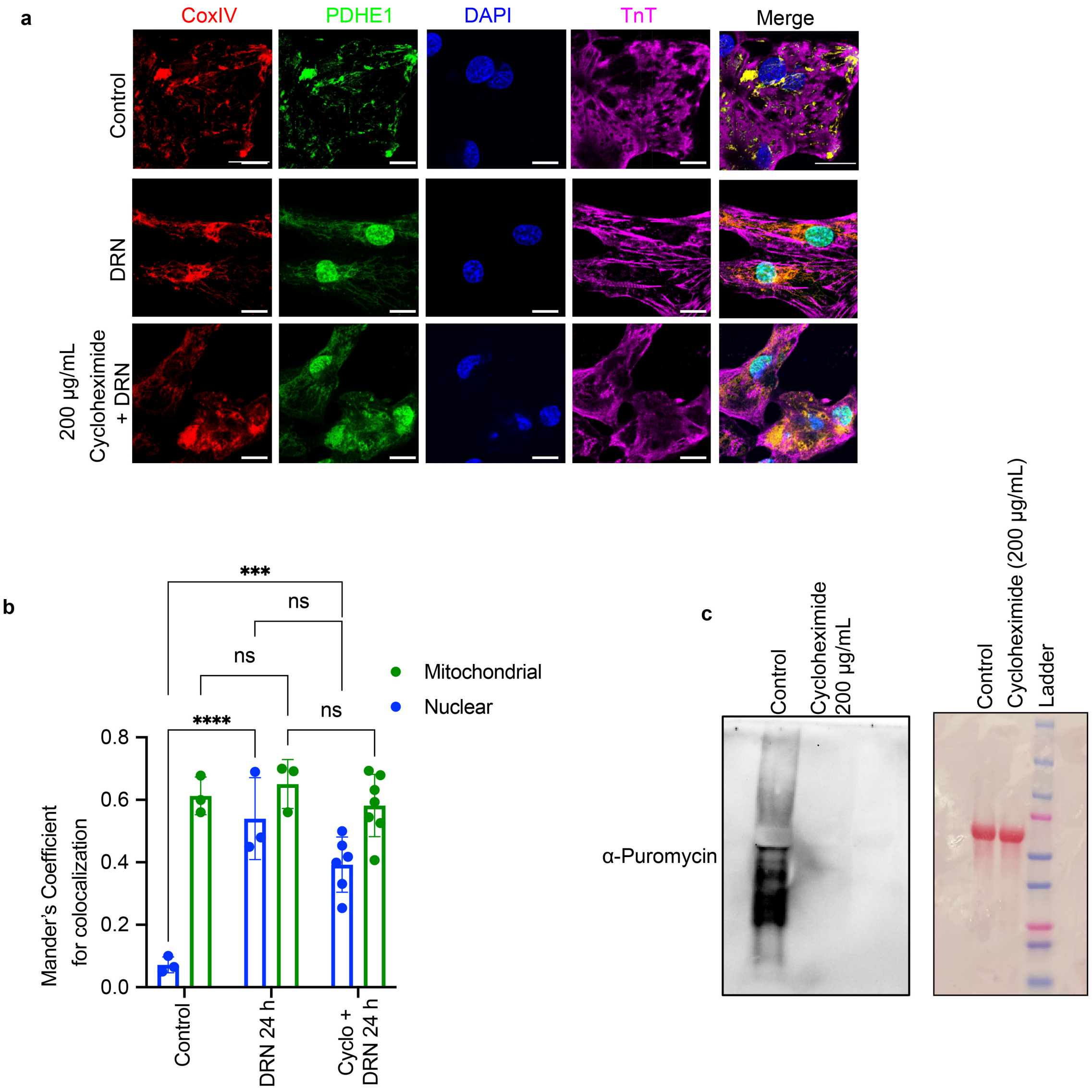

Supplementary Fig.6: (a-c) confocal images showing the translocation of PDH-E1 to the nuceli in presence of cycloheximide in DRN-treated cells **(a)**, quantification of images shown in panel a **(b)**, and sunset assay measuring the incorporation of puromycin in cycloheximide treated cells as active translation as an effect of cycloheximide on protein synthesis **(c)**. Scale bar. 20 µm. p-value**** >0.0001, p-value***=0.0009

Supplementary Fig.7

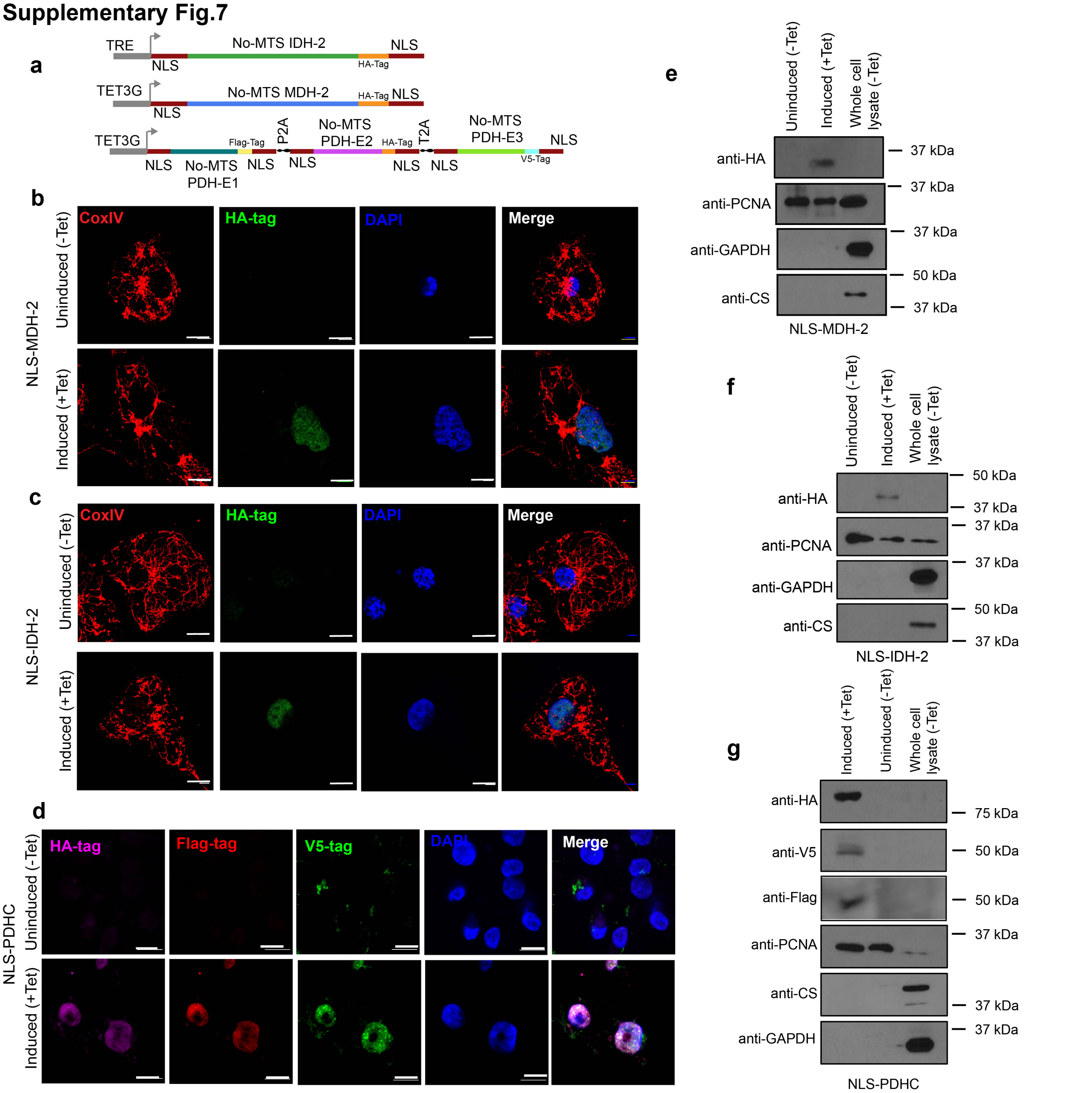

Supplementary Fig.7: (a) Construct design of plasmid inserts for tetracycline-inducicble nuclear translocation of TCA cycle enzymes. **(b-d)** Confocal microscopy images of iPSC-derived cardiomyocytes transduced with lentiviruses carrying tetracycline-inducible nuclear targeting MDH-2 **(b)**, IDH-2 **(c)**, and PDHC **(d)**. The cells were stained with anti-HA-Tag for MDH-2 and IDH-2, and with anti-HA-tag, anti-Flag tag and anti-V5 for PDHC to differentiate the mitochondrial pool of the proteins. Images were background corrected based on non-transduced cells stained with same antibodies. **(e-g)** Western blots of nuclear lysates showing tetracycline-induced nuclear translocation of MDH-2 **(e)**, IDH-2 **(f)**, and PDHC **(g)**. Blots were probed for GAPDH as cytoplasmic control, CS was probed as mitochondrial control and PCNA as loading control for nuclear fractions. Whole cell lysate from uninduced cells was used as another control, the detection of GAPDH and CS only in the whole cell lysate and not in the nuclear fractions shows the purity of nuclear fractions.

Supplementary Fig.8

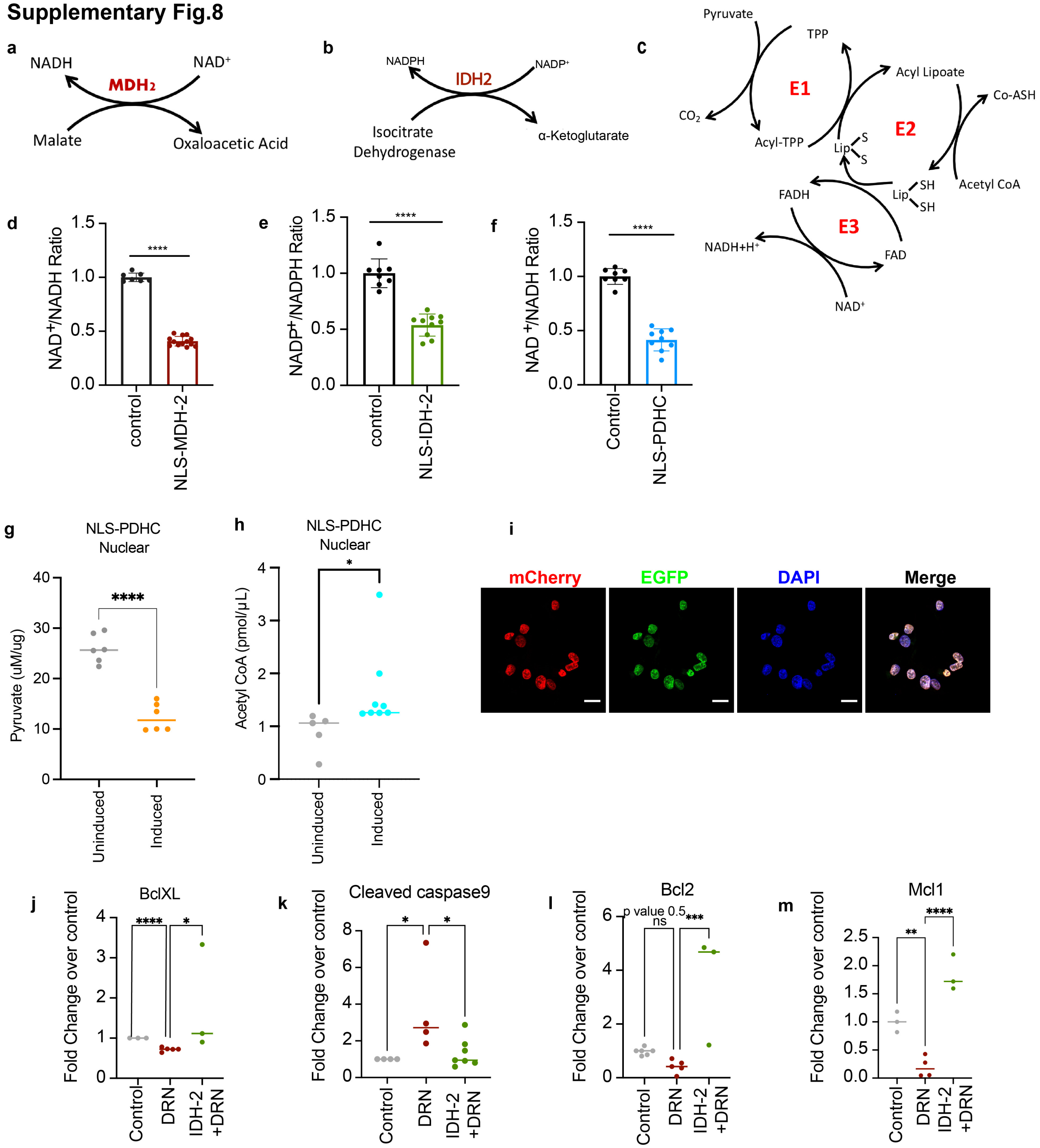

Supplementary Fig.8: (a-f) Reaction catalyzed by MDH-2 **(a)**, IDH-2 **(b)**, and PDHC **(c)** detected to measure the enzyme activity of these enzymes shown in figure 2a-c. **(d-f)** $NAD^+/NADH$ or $NADP^+/NADPH$ ratio in nuclei isolated from cells transduced with nuclear targeting MDH-2 **(d)**, IDH-2 **(e)**, and PDHC **(f)** viruses. p-value****<0.0001, **(g-h)** Nuclear levels of Pyruvate **(g)** and AcetylCoA **(h)** in nuclei isolated from cells expressing NLS-PDHC. p-value****<0.0001, p-value*=0.0342. **(i)** Image of iPSC-CM expressing NLS-EGFP-2A-NLS-mCherry . scale bar 20 μm **(j-m)** quantification of western blots shown in figure 2. p value****<0.0001, p value*<0.05, p value***=0.001, p value**=0.005,

Supplementary Fig.9

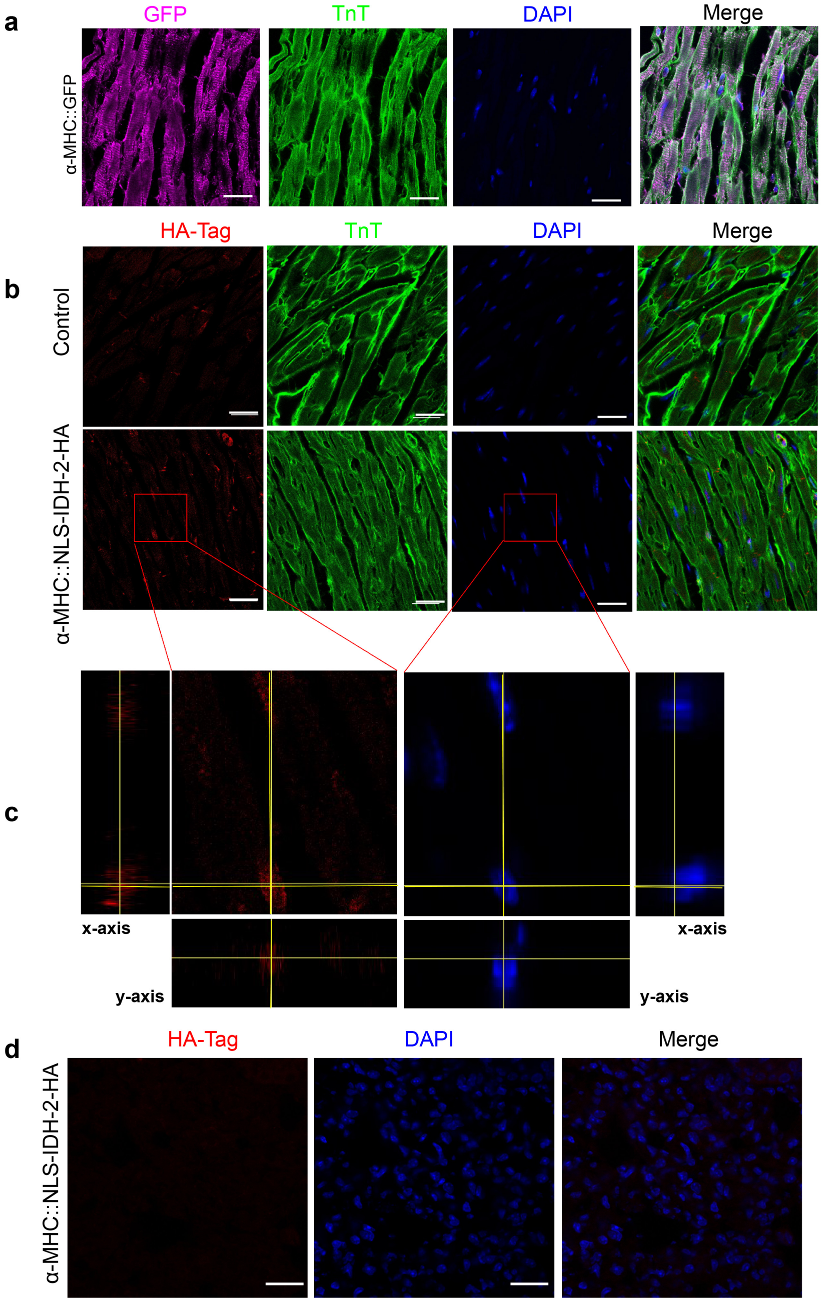

Supplementary Fig.9: (a) Heart section from mouse injected with α-MHC::EGFP showing expression of EGFP (in magenta) in cardiomyocytes counterstained with Troponin T (TnT in gree). (b) Heart section from mouse injected with α-MHC::NLS-IDH-2-HA showing expression of HA tagged IDH-2 in nuclei (Blue) of cardiomyocytes (Red) counterstained with Troponin T (TnT in green) scale bar 10 μm . (c) Orthogonal section of enlarged field corresponding to sections selectesd from panel b showing the HA-Tag (red) colocalizes with nucleus (Blue). (d) Lung section from mouse expressing α-MHC::NLS-IDH-2-HA, the section was stained for HA-tag, no HA-Tag staining shows cardiac-specific expression of NLS-IDH-2-HA when expressed under α-MHC promoter. scale bar 10 μm

Supplementary Fig.10

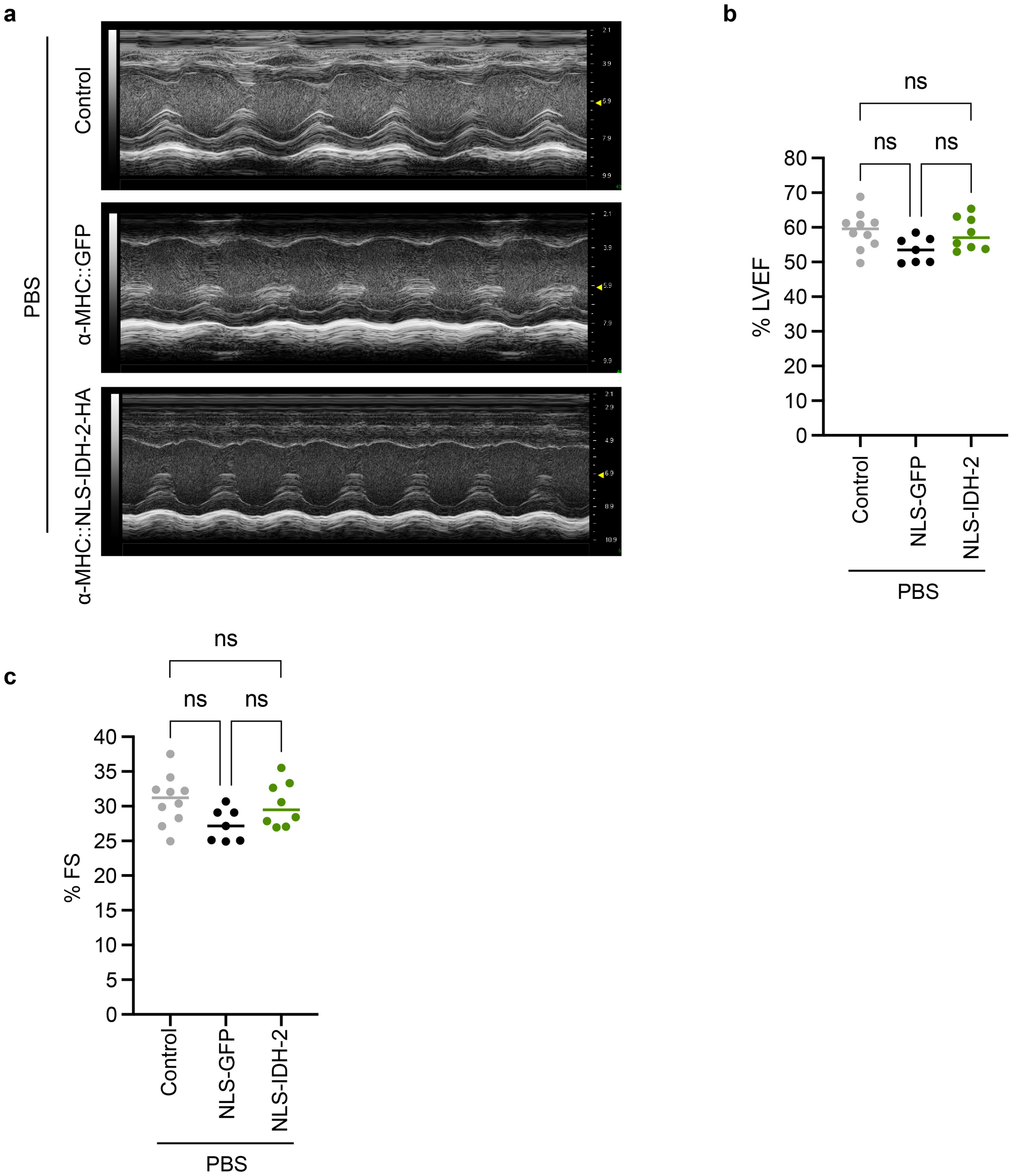

Supplementary Figure 10. (a-c) M-mode of ECHO images (a), and LVEF (b) and FS (c) of mice injected with no virus or α-MHC-NLS-GFP or α-MHC-NLS-IDH-2.

Supplementary Fig.11

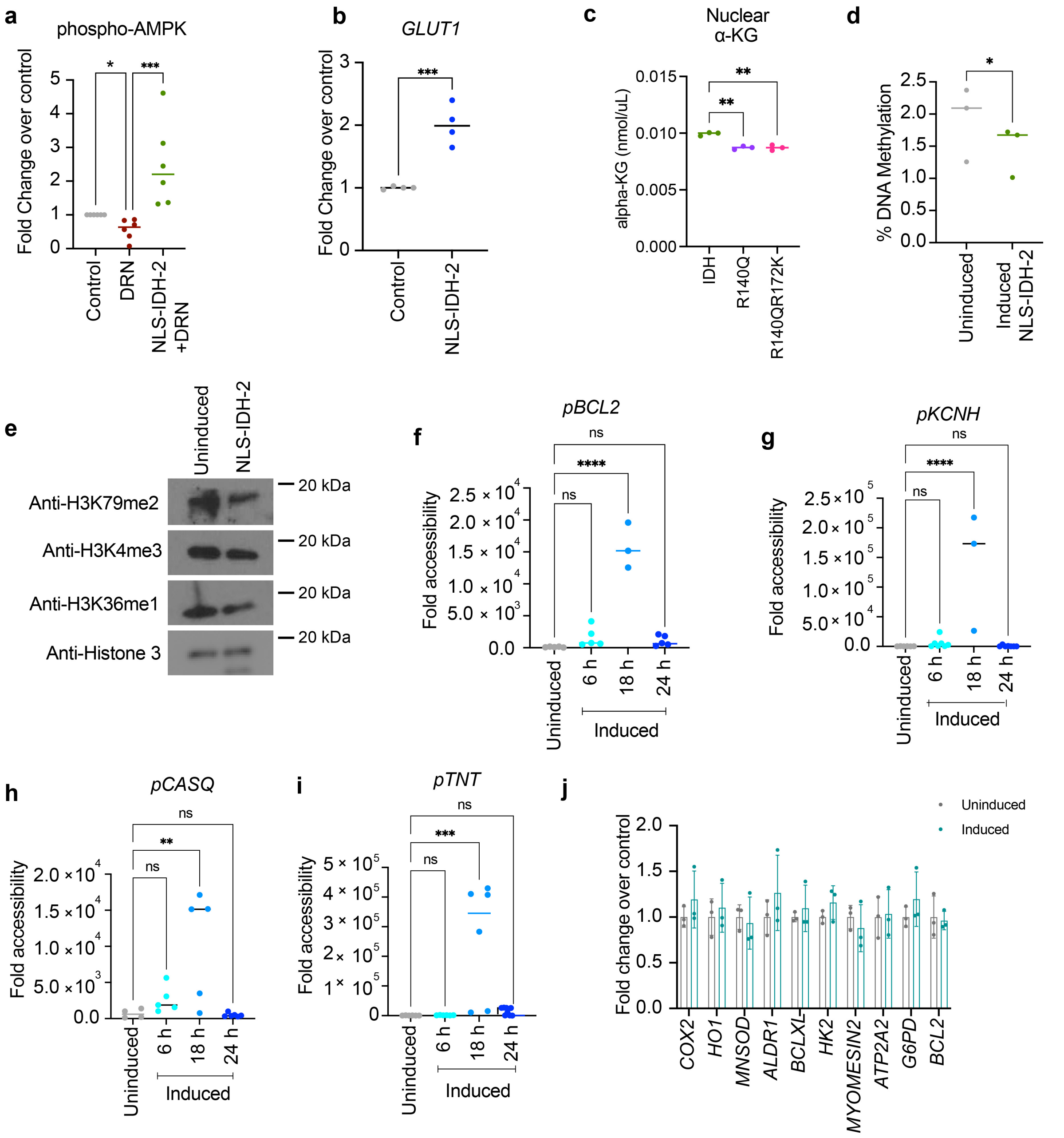

Supplementary Fig.11: (a) quantification of blots shown in figure 4 a p-Value*<0.05, ***<0.001. **(b)** mRNA levels of downstream target of AMPK activation, p value***=0.0008. **(c)** nuclear α-KG levels in nuclei isolated from cells expressing IDH-2 mutants in the nuclei of iPSC-CMs. p value**<0.01,**(d)** Level of gDNA methylation in cells expressing nuclear IDH-2 p-Value*=0.0341. **(e)** Western blots showing histone methylation status in cells expressing nuclear IDH-2. **(f-i)** ATAC-qPCR of *pBCL2*, *pKCNH, pCASQ, pTNT* showing accessibility of promoter region 6h, 18h, and 24h post tetreacycline induction of nuclear expression of IDH-2. p value****<0.0001, p value ***0.0003, p value =0.0054. **(j)** mRNA levels of target genes for ATAC-qPCR18 h post tetracycline induction of nuclear expression of IDH-2.

Supplementary Fig.12

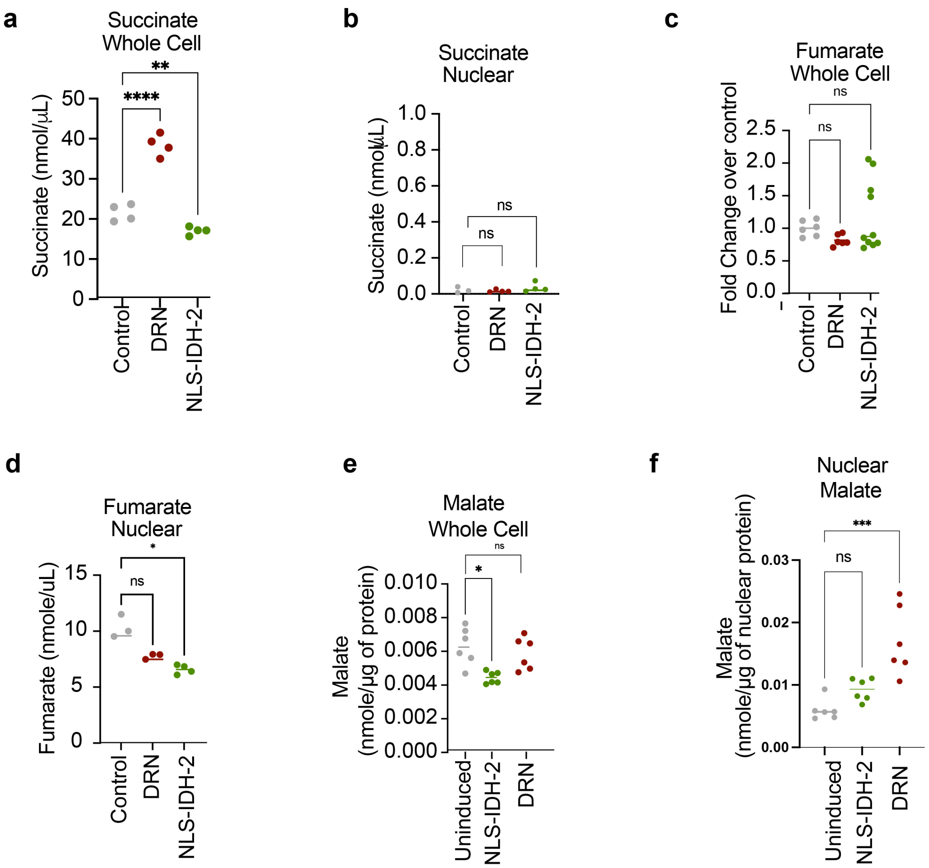

Supplementary Fig.12: (a-b) whole cell **(a)** and nuclear levels **(b)** of Succinate, **(c-d)** whole cell **(c)** and nuclear levels **(d)** of Fumarate, **(e-f)** whole cell **(e)** and nuclear levels **(f)** of Malate. p-value**** <0.0001, **<0.01, *<0.05, ***=0.0001

Supplementary Fig.13

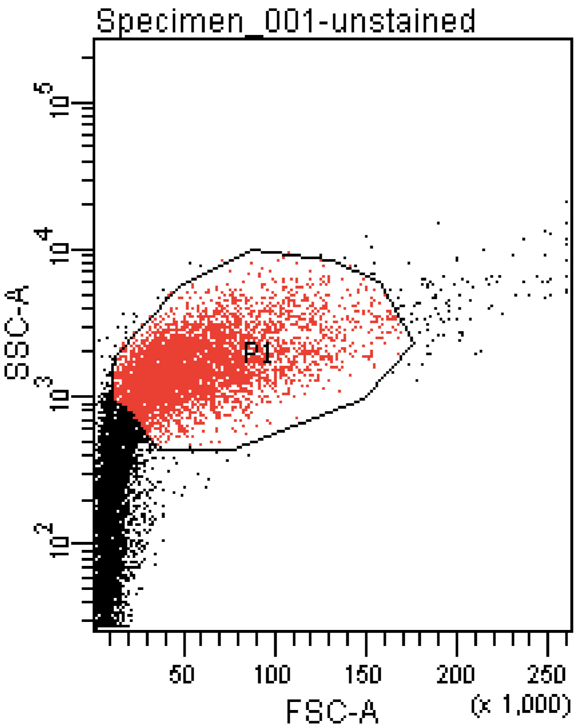

Supplementary Fig.13. Representative image of gating strategy in unstained cells to exclude debris and doublet cells in flow cytometry experiments using annexinV-FITC.

Supplementary Table. 1. Primer list for ATAC-qPCR

Target	Orientation	Sequence
pTNT	Forward	AAAGGACATCCTGGGCTGGGAGCTG
	Reverse	ACTGCGCGATTGCAGGCCAATTAGT
pKCNH2	Forward	CAGGCTGCTGCCCACGCTTACTG
	Reverse	AGAGCCCCTGTCCTGCTCGCCTTC
pRYR2	Forward	CGAGGGCGAAGACGAGATCCAGTTC
	Reverse	AGCCGCGCTCCATCCTCTAGCTTTC
pCASQ	Forward	CCCTTGGCTCTGTCGGCAGTTTCTC
	Reverse	GGTGTCCCTAGCACCAGCAGCAACA
pBCL2	Forward	CTCCCGAGCCTCTGGGGTACAGCTT
	Reverse	TACCGCTCAGCACCTGTCCGGTTTC
pADLRED	Forward	CCTTTCTGCCGACCTCACGGGCTAT
	Reverse	GGGCGAGCTGCTCAGGGTCACTC
pATP2A2	Forward	TGTGCAGACAGCTATTTCGCACTGT
	Reverse	GTCCCCCTCCCACAGAACAGTCACC
pBCLXL	Forward	AGCCAAGGGGCGTGCAAGAGAGAG
	Reverse	TGCAGCCCCCGGAAGATCTTTTGTA
pCOX-2	Forward	GGGGAGGAGAGGGAGGGATCAGACA
	Reverse	GCTCCTGACGCTCACTGCAAGTCGT
pG6PD	Forward	GGTGTCATAGCTGTGGGATCCGGAAGT
	Reverse	CCGGCCCTATTGGGCAGTCTCTTCT
pHEMOXIGENASE 1	Forward	CCCAGAGCCTGCAGCTTCTCAGATT
	Reverse	GTCACATTTATGCTCGGCGGGTCAC
pHEXOKINASE 2	Forward	CGGCAGCCCCTCAATAAGCCACATT
	Reverse	CCTCAGTTCTCTCCTGGCGACGTGT
pSOD2	Forward	AAGTACGGCAGACAGGCAGCGAGGT
	Reverse	GAAGCCACCACAGCCACGAGTGC
pMYOMESIN	Forward	CTCTTTCTGGGTCGGGGGCTGAAGT
	Reverse	GGCAGAGCTGACTCCTGCAAAGCAA

Supplementary Table. 2. Primers for mRNA-qPCR

Target	Orientation	Sequence
TNT	Forward	GGAGAGAGAGTGGACTTTGATG
	Reverse	CCTCCTCTTTCTTCCTGTTCTC
KCNH2	Forward	CGCTGTTATCACGCAAACTATG
	Reverse	CGGATGGGAGTTGGTCATTTA
RYR2	Forward	GAACGAGAGGTCAGCGAATAAG
	Reverse	ACCGTCAGAATGGAGAAGAAAG
CASQ	Forward	CCCAATAGCGAAGAGGAGATTG
	Reverse	TCCTCCCAGGTCTCATACATAC
BCL2	Forward	GATTGTGGCCTTCTTTGAG
	Reverse	GTTCCACAAAGGCATCC
ADLRED	Forward	CCAACTTCAACCATCTCCAGGTG
	Reverse	GTCACCACGATGCCTTTGGACT

ATP2A2	Forward	GGACTTTGAAGGCGTGGATTGTG
	Reverse	CTCAGCAAGGACTGGTTTTCGG
BCLXL	Forward	GCCACTTACCTGAATGACCACC
	Reverse	AACCAGCGGTTGAAGCGTTCCT
COX-2	Forward	CGGTGAAACTCTGGCTAGACAG
	Reverse	GCAAACCGTAGATGCTCAGGGA
G6PD	Forward	TGCCTCCACTTTACCAGCTACGTT
	Reverse	TCTCATTTGTCGTCCGGTCACTGT
HEMOXIGENASE 1	Forward	CCAGGCAGAGAATGCTGAGTTC
	Reverse	AAGACTGGGCTCTCCTTGTTGC
HEXOKINASE 2	Forward	GAGTTTGACCTGGATGTGGTTGC
	Reverse	CCTCCATGTAGCAGGCATTGCT
SOD2	Forward	CTGGACAAACCTCAGCCCTAAC
	Reverse	AACCTGAGCCTTGGACACCAAC
MYOMESIN	Forward	CTACCTGGACAAGCGTGAAGTTC
	Reverse	CGTAGAGTGAGCCTTCCGTCAA
GLUT1	Forward	GATGCGGGAGAAGAAGGTCA
	Reverse	TAGAAGACAGCGTTGATGCCA
MCL1	Forward	TAGTTAAACAAAGAGGCTGG
	Reverse	ATAAACTGGTTTTGGTGGTG
GAPDH	Forward	CGGCGCAGCAGTGAAAGGGAGT
	Reverse	TAGGGCAGACAATCCCGGCCC
BETA ACTIN	Forward	AGAAAATCTGGCACCACACC
	Reverse	CCATCTCTTGCTCGAAGTCC
