# Supplementary material for: Nuclear translocation of mitochondrial dehydrogenases as an adaptive cardioprotective mechanism
Source: Nat Commun. 2023 Jul 19;14:4360. doi: 10.1038/s41467-023-40084-5 (PMC10356764; doi:10.1038/s41467-023-40084-5)
Supplement: Supplementary file 3 — Description of Additional Supplementary Files [file 41467_2023_40084_MOESM3_ESM.pdf]

Description of Additional Supplementary Files

Supplementary Movie 1: MDH-2 staining in DMSO-treated control iPSC cardiomyocytes. Video representing 3-dimensional reconstitution of confocal z-stacks of control iPSC cardiomyocytes showing co-localization of **MDH-2** (green) with Cox-IV (red), which was counterstained for mitochondria (represented in figure 1 C). The image suggests that in control cells, MDH-2 localizes completely in the mitochondria, not in the nucleus, stained with DAPI (blue). Scale bar: 10 µm.

Supplementary Movie 2: MDH-2 staining in doxorubicin-treated iPSC cardiomyocytes. Video representing 3-dimensional reconstitution of confocal z-stacks of doxorubicin-treated iPSC cardiomyocytes showing localization of **MDH-2** (green) with both mitochondria (red) and nuclei (blue) (represented in Figure 1 D). CoxIV was used to counterstain mitochondria, and DAPI was used as a nuclear stain. Scale bar: 10 µm.

Supplementary Movie 3: IDH-2 staining in DMSO-treated control iPSC cardiomyocytes. Video representing 3-dimensional reconstitution of confocal z-stacks of control iPSC cardiomyocytes showing co-localization of **IDH-2** (green) with Cox-IV (red), which was counterstained for mitochondria. Image suggests that in control cells, IDH-3 localizes completely in the mitochondria not in the nucleus stained with DAPI (blue) (Represented in figure 1 E). Scale bar: 10 µm.

Supplementary Movie 4: IDH-2 staining in doxorubicin-treated iPSC cardiomyocytes. Video representing 3-dimensional reconstitution of confocal z-stacks of doxorubicin-treated iPSC cardiomyocytes showing localization of **IDH-2** (green) with both mitochondria (red) and nuclei (blue) (represented in Figure 1 F). CoxIV was used to counterstain mitochondria, and DAPI was used as a nuclear stain. Scale bar: 10 µm.

Supplementary Movie 5: IDH-2 staining in cardiomyocytes isolated from control mice. Video representing 3-dimensional reconstitution of confocal z-stacks of cardiomyocytes isolated from control mice showing localization of IDH-2 (green) outside the nucleus (blue). DAPI was used as a nuclear stain. Scale bar: 10 µm.

Supplementary Movie 6: IDH-2 staining in cardiomyocytes isolated from doxorubicin-treated mice. Video representing 3-dimensional reconstitution of confocal z-stacks of cardiomyocytes isolated from doxorubicin-treated mice showing localization of IDH-2 (green) both outside and inside the nucleus (blue). DAPI was used as a nuclear stain. Scale bar: 5 µm.

Supplementary Movie 7: IDH-2 staining in cardiac tissue sections isolated from PBS-treated mice. Video showing that in PBS treated mice, the IDH-2 (green) resides outside the nucleus (DAPI). TroponinT (magenta) was probed to counterstain cardiomyocytes. Scale bar: 5 µm.

Supplementary Movie 8: IDH-2 staining in cardiac tissue sections isolated from DRN-treated mice. Video showing that in DRN treated mice, the IDH-2 (green) resides both outside the nucleus and also co-localizes with the nucleus (DAPI) in the cardiomyocytes (magenta). TroponinT (magenta) was probed to counterstain cardiomyocytes. Scale bar: 5 μm.
